# Comparing SARC-CalF With SARC-F for Screening Sarcopenia in Adults With Type 2 Diabetes Mellitus

**DOI:** 10.3389/fnut.2022.803924

**Published:** 2022-03-31

**Authors:** Zeru Xu, Ping Zhang, Yifei Chen, Jiahong Jiang, Zijun Zhou, Hong Zhu

**Affiliations:** ^1^Department of Endocrinology and Metabolism, The First Affiliated Hospital of Wenzhou Medical University, Wenzhou, China; ^2^Department of Endocrinology, The Second People’s Hospital of Xining, Xining, China; ^3^Department of Medicine, Changsha Medical University, Changsha, China

**Keywords:** type 2 diabetes mellitus, sarcopenia, SARC-F, SARC-CalF, specificity, sensitivity, diagnostic criteria

## Abstract

**Background:**

The prevalence of sarcopenia is high in older people with type 2 diabetes mellitus (T2DM) and is now considered a critical problem in the healthcare sector. However, the preferred screening tool for identifying sarcopenia remains unknown. Thus, the aim of this study was to ensure that the diagnostic values of the SARC-F (strength, assisting with walking, rising from a chair, climbing stairs, and falling) and SARC-CalF (SARC and calf circumference) scales were compared with five reference diagnostic criteria for sarcopenia.

**Methods:**

This was a cross-sectional study. Patients diagnosed with diabetes were treated at the First Affiliated Hospital of Wenzhou Medical University. Appendicular skeletal muscle mass, muscle strength, and physical performance were assessed using dual-energy X-ray absorptiometry, handgrip strength, and gait speed assessment. Five diagnostic criteria for sarcopenia (Asian Working Group for Sarcopenia, International Working Group on Sarcopenia, Foundation for the National Institutes of Health, Sarcopenia Project, Society on Sarcopenia Cachexia and Wasting Disorders, and European Working Group on Sarcopenia in Older People criteria) were utilized. Sensitivity and specificity analyses were performed on the SARC-CalF and SARC-F scales. The diagnostic precision of both instruments was determined using the receiver-operating characteristic (ROC) curves and area under the ROC curves (AUC).

**Results:**

This study included 689 subjects (459 men and 230 women) with a mean age of 58.1 ± 13.2 years. In accordance with the five reference diagnostic parameters, the prevalence of sarcopenia was between 4.5 and 19.2%. In addition, the range of sensitivity of SARC-F and SARC-CalF ranged from 61.4 to 67.4 and 82.6 to 91.8%, respectively. Concurrently, the specificity ranged from 63.1 to 67.3 and 51.5 to 61.2%, respectively. Overall, AUC values for SARC-CalF were higher than those for SARC-F, regardless of the diagnostic standard, sex, or age.

**Conclusion:**

The results of this study suggest that SARC-CalF significantly enhances the sensitivity and overall diagnosis of SARC-F. SARC-CalF appears to be an optimal screening tool for sarcopenia in adults with T2DM.

## Introduction

According to the definition, sarcopenia is the accelerated loss of skeletal muscle mass, muscle strength, and physical performance associated with old age or chronic disease. Additionally, it can result in negative effects, including falls, fractures, functional disability, enhanced hospital admission rates, reduced quality of life, and even death (1).

Diabetes mellitus (DM) is a metabolic disease that lasts for a lifetime. As individuals suffering from diabetes live longer, sarcopenia has recently been considered as one of the chronic complications of DM (1). Various studies have indicated that the prevalence of sarcopenia in people with type 2 DM (T2DM) is 1.56–3 times higher than in non-diabetics. In addition, sarcopenia is significantly related to glycemic control and the duration of diabetes (2–4). Therefore, early detection, early diagnosis, and treatment of sarcopenia in patients with diabetes are highly crucial. Current guidelines allow certain devices to evaluate body composition, such as computed tomography (CT), dual-energy X-ray absorption (DXA), and bioimpedance analysis (BIA). Nevertheless, since these devices are inaccessible in many clinical situations, a short and easy-to-use sarcopenia screening tool is needed (5, 6).

Several screening tools are available for screening sarcopenia. For instance, SARC-F, which was developed in 2013, is the most extensively applied questionnaire in several populations. SARC-F focuses on strength, assisting with walking, rising from a chair, climbing stairs, and falling (7). However, it has shown high specificity but low sensitivity, which may affect its potential as a screening tool for identifying individuals with sarcopenia (8–10). Recently, another tool for screening sarcopenia, known as SARC-CalF (SARC and calf circumference) (11), was reported by Sliva et al. This integrates SARC-F and calf circumference (CC), which greatly enhances the sensitivity and accuracy of SARC-F diagnosis. Yang et al. equally discovered similar results in Chinese elderly people (12, 13). Nevertheless, these results should be applied to diverse populations.

From what is known, people with diabetes have a higher tendency for developing sarcopenia; however, few studies have examined the diagnostic performance of SARC-F and SARC-CalF in predicting sarcopenia in adults with T2DM. Therefore, a cross-sectional study was performed to fill this gap.

## Materials and Methods

### Study Design and Population

In this study, a diagnostic accuracy study was conducted. From June 2020 to June 2021, a total of 689 inpatients with T2DM according to the criteria of the American Diabetes Association of the Endocrinology Department were recruited at the First Affiliated Hospital of Wenzhou Medical University, Wenzhou, China. The exclusion criteria were as follows: (1) age ≤18 years; (2) type 1 DM (T1DM) and other types of DM; (3) malignant tumor; (4) autoimmune diseases; (5) taking medications that may affect body composition; (6) long-term bedridden patients; (7) severe disease of the heart, liver, or kidneys; and (8) inability to communicate with investigators.

All participants provided written informed consent. The study protocol utilized in this study was approved through the Clinical Research Ethics Committee of the First Affiliated Hospital of Wenzhou Medical University.

### Assessment of Sarcopenia With SARC-F and SARC-CalF

To evaluate sarcopenia risk, the SARC-F and SARC-CalF scales were implemented (Supplementary Table 1).

#### The SARC-F Scale

The SARC-F scale examines five domains: (1) strength; (2) walking assistance; (3) rising from a chair; (4) climbing stairs; and (5) falls, with scores ranging between 0 and 2. A score of ≥4 points implies positive sarcopenia screening (7).

#### The SARC-CalF Scales

The SARC-CalF scale contains six objects; the first five objects have the same score as SARC-F, and the sixth object is CC. In accordance with the 2019 Asian Working Group for Sarcopenia (AWGS) criteria, CC thresholds were 34 and 33 cm for men and women, respectively. If the score is above the cut-off value, CC is scored as 0; if it is below the cut-off value, the score is 10. A score of ≥11 points indicates a positive screening for sarcopenia (5).

### Assessment of Muscle Mass, Muscle Strength, and Physical Performance

Muscle mass was assessed in each subject using DXA (Model: Prodigy Primo – 81013GA series; software 11.40.004, GE Healthcare United States, Shanghai Agent in Asia, Shanghai, China) by experienced radiologists. Before analysis, subjects were required to wear only a hospital gown and all-metal accessories. Scanning was taken while lying down. The software provides estimates of appendicular skeletal muscle mass (ASM), which is the sum of lean body mass in the upper and lower extremities (14). The ASM assessed and calculated the skeletal muscle mass index (SMI) and ASM/body mass index (BMI). The SMI was computed from the equation: $\mathrm{SMI}\,\left(\text{kg}/{\text{m}}^{2}\right)=\frac{\mathrm{ASM}\,\left(\mathrm{kg}\right)}{{\text{height}}^{2}\,\left({\text{m}}^{2}\right)}$.

The muscle strength was assessed on the basis of handgrip strength (HGS) using a portable electronic dynamometer with a precision of 0.1 kg (Brand: CAMRY, Model: TH-01, XIANGSHAN, Zhongshan, and Guangdong Province, China). Subjects who performed the HGS test were seated with their arms at their sides and with their elbows flexed at 90°. They squeezed the handle as hard as they could. Trained surveyors assessed the subject’s dominant hand three times, with an interval of 1 min between each measurement. All measured values were recorded, and the maximum value was taken as the muscle strength.

In this study, the gait speed (GS) test was measured as the physical performance. The subjects were instructed to walk a distance of 6 m in their normal gait. If necessary, canes and walkers were adopted. The GS measurement was performed twice with an accuracy of 0.1 m, and the mean value was recorded.

### Clinical Data

General clinical data, such as demographic information, duration of illness, medical history, and medication status, were collected.

All anthropometric measurements were performed in the morning without eating before the assessments. Trained nurses measured weight (kg) and height (m) at 0.1 kg and 0.1 cm, respectively. BMI (kg/m^2^) was computed as the body weight divided by the square of the height. The CC was assessed when the subjects were in a sitting position with their soles touching the surfaces of the floor, with the assumption that the widest circumference of the right calf was being used. Waist circumference (WC) was measured midway between the top of the hip bone and the lower rib when the subjects were in a standing position. The measurement of the CC and WC required the use of anthropometric tape. CC and WC must be accurate to the nearest 0.1 cm.

After fasting overnight, blood samples were taken from the antecubital vein and centrifuged at 5000 rpm for 20 min. Before conducting the assay, the plasma was stored in freezing tubes at −80°C. Biochemical indices include glycated hemoglobin (HbA1c), fasting plasma glucose (FPG), 2-h postprandial glucose (2hPG), alanine aminotransferase (ALT), and aspartate aminotransferase (AST). All items were assessed in the Biochemistry Department of the First Affiliated Hospital of Wenzhou Medical University. Insulin resistance (IR) was assessed through the application of the homeostasis model (HOMA-IR), which was computed as follows: $\text{HOMA}-\text{IR}=\frac{\text{FPG}\,{\left(\text{mmol}/\text{l}\right)}^{*}\,\text{FINS}\,\left(\text{mIU}/\text{l}\right)}{22.5}$ (15), and the estimated glomerular filtration rate (eGFR) was computed in accordance with the CKD-EPI formula (16).

### Assessment of Sarcopenia Using Different Diagnostic Criteria

The five diagnostic classifications are utilized for screening sarcopenia: (1) the European Working Group on Sarcopenia in Older People (EWGSOP) (17); (2) the AWGS (5); (3) the International Working Group on Sarcopenia (IWGS) (18); (4) the Society on Sarcopenia Cachexia and Wasting Disorders (SCWD) (19); and (5) the Foundation for the National Institutes of Health (FNHI) Sarcopenia Project (20).

Sarcopenia was defined according to the EWGSOP1 and AWGS2019 criteria as low muscle mass, in addition to low muscle strength or low physical performance. According to the IWGS and SCWD criteria, sarcopenia is defined as low muscle mass and poor physical performance. According to the FNHI recommendation, sarcopenia is defined as a low muscle mass associated with low muscle strength. Various diagnostic criteria recommended different cut-off values and were inconsistent for both sexes. The comprehensive criteria used in this study are listed in Table 1.

**TABLE 1:** Five diagnostic criteria for sarcopenia and the cut-off applied.

Diagnosis definition	Muscle mass	Muscle strength	Physical performance
EWGSOP1	SMI ≤ 7.26 kg/m^2^ for men SMI ≤ 5.50 kg/m^2^ for women	HGS < 30 kg for men HGS < 20 kg for women	GS < 0.8 m/s for both gender
AWGS2019	SMI < 7 kg/m^2^ for men SMI < 5.4 kg/m^2^ for women	HGS < 28 kg for men HGS < 18 kg for women	GS < 1 m/s for both gender
IWGS	SMI ≤ 7.2 kg/m^2^ for men SMI ≤ 5.67 kg/m^2^ for women		GS < 1 m/s for both gender
SCWD	SMI ≤ 6.81 kg/m^2^ for men SMI ≤ 5.18 kg/m^2^ for women		GS < 1 m/s for both gender
FNIH	ASM/BMI < 0.789 for men ASM/BMI < 0.512 for women	HGS < 26 kg for men HGS < 16 kg for women	

*SMI, skeletal muscle mass index; ASM, appendicular skeletal muscle mass; BMI, body mass index; HGS, handgrip strength; GS, gait speed.*

### Statistical Analysis

SPSS software (version 23.0; SPSS Statistics, IBM, Armonk, NY, United States) and MedCalc statistical software (version 19.0; MedCalc software bvba, Ostend, Belgium) were used for statistical analysis. A two-tailed *p*-value of <0.05 was considered to indicate significant differences.

The clinical properties of the substances between men and women were compared. Continuous variables with a normal distribution were presented as mean ± SD. For continuous variables with skewed distributions, data were presented as the median (interquartile range). Student’s *t*-test and Mann–Whitney *U*-test were used to compare continuous variables. For categorical variables, data were presented as numbers (percentage). The *X*^2^ test was used to compare the categorical variables.

The EWGSOP1, AWGS2019, IWGS, SCWD, and FNIH were used as reference standards. The diagnostic value was calculated, for instance, sensitivity, specificity, positive predictive value (PPV), negative predictive value (NPV), positive likelihood ratio (+LR), and negative likelihood ratio (−LR) for SARC-F and SARC-CalF to screen for sarcopenia. The receiver-operating characteristic (ROC) curve was used to compare the overall diagnostic precision and calculate the area under the ROC curves (AUC) at a 95% confidence interval (CI). Overall, AUCs <0.5, 0.5–0.7, 0.7–0.9, and >0.9 demonstrate that a tool has no, low, moderate, and high diagnostic values (21). The AUC values for each ROC curve were compared using the DeLong method (22). The data were also stratified by age and sex to investigate the capacity and application of the two screening tools.

## Results

### Subjects’ Description and Characteristics

In total, 689 T2DM inpatients from our clinic were available for assessment. Regarding the population sample, there are about 459 men and 230 women with a mean age of 56.4 ± 13.6 years for men and 61.7 ± 11.7 years for women. The properties of the substances according to sex are summarized in Table 2. In accordance to the table, women had a higher mean age than men (*p* < 0.001), duration of diabetes (*p* = 0.028), 2hPG (*p* = 0.023), HOMA-IR (*p* < 0.001), SARC-F scores (*p* < 0.001), and SARC-ClaF scores (*p* < 0.001). In contrast, men had considerably higher levels of ALT (*p* < 0.001), AST (*p* = 0.007), CC (*p* < 0.001), HGS (*p* < 0.001), GS (*p* < 0.001), SMI (*p* < 0.001), and ASM/IMC (*p* < 0.001). Additionally, FPG, eGFR, BMI, and WC were not significantly different between the two groups.

**TABLE 2:** Characteristics of the study population.

Variable	Overall (*n* = 689)	Men (*n* = 459)	Women (*n* = 230)	*p*-Value^a^
Age (years)*	58.1 ± 13.2	56.4 ± 13.6	61.7 ± 11.7	<0.001
Diabetes duration (years)^‡^	10.0 (3.0, 15.0)	9.0 (2.0, 15.0)	10.0 (5.0, 15.3)	0.028
HbA1c (%)*	9.6 ± 2.4	9.7 ± 2.4	9.3 ± 2.3	0.040
FPG (mmol/L)*	8.9 ± 3.0	8.8 ± 2.8	9.1 ± 3.2	0.206
2hPG (mmol/L)*	19.8 ± 4.7	19.5 ± 4.6	20.4 ± 4.9	0.023
HOMA-IR^‡^	2.7 (1.7, 4.6)	2.5 (1.5, 3.9)	3.4 (1.9, 5.2)	<0.001
ALT (U/L)^‡^	21.0 (14.0, 32.0)	23.0 (15.0, 35.0)	17.0 (13.0, 25.0)	<0.001
AST (U/L)^‡^	21.0 (17.0, 29.0)	22.0 (17.0, 31.0)	20.0 (17.0, 26.0)	0.007
eGFR*	95.2 ± 28.3	96.4 ± 30.6	92.8 ± 22.8	0.112
BMI (kg/m^2^)*	23.6 ± 3.4	23.7 ± 3.4	23.2 ± 3.3	0.076
WC (cm)*	90.1 ± 9.4	90.5 ± 9.4	89.3 ± 9.4	0.133
CC (cm)*	33.7 ± 3.6	34.3 ± 3.6	32.5 ± 3.2	<0.001
HGS (kg)*	29.0 ± 10.5	33.6 ± 9.3	19.8 ± 5.8	<0.001
GS (m/s)*	1.0 ± 0.2	1.0 ± 0.2	0.9 ± 0.2	<0.001
ASM (kg)*	20.0 ± 4.4	22.1 ± 3.6	15.9 ± 2.6	<0.001
SMI (kg/m^2^)*	7.3 ± 1.2	7.7 ± 1.1	6.4 ± 0.9	<0.001
ASM/BMI*	0.9 ± 0.2	0.9 ± 0.1	0.7 ± 0.1	<0.001
SARC-F^‡^	0 (0, 1)	0 (0, 1)	1 (0, 1)	<0.001
SARC-CalF^‡^	3 (0, 10)	1 (0, 10)	10 (1, 11)	<0.001
**SARC-F classification^†^**				0.070
Non-sarcopenia	658 (95.5%)	443 (96.5%)	215 (93.5%)	
Sarcopenia	31 (4.5%)	16 (3.5%)	15 (6.5%)	
**SARC-CalF classification^†^**				<0.001
Non-sarcopenia	549 (79.7%)	386 (84.1%)	163 (70.9%)	
Sarcopenia	140 (20.3%)	73 (15.9%)	67 (29.1%)	
**EWGSOP1 classification^†^**				0.063
Non-sarcopenia	557 (80.8%)	362 (78.9%)	195 (84.8%)	
Sarcopenia	132 (19.2%)	97 (21.1%)	35 (15.2%)	
**AWGS2019 classification^†^**				0.243
Non-sarcopenia	574 (83.3%)	377 (82.1%)	197 (85.7%)	
Sarcopenia	115 (16.7%)	82 (17.9%)	33 (14.3%)	
**IWGS classification^†^**				0.732
Non-sarcopenia	607 (88.1%)	403 (87.8%)	204 (88.7%)	
Sarcopenia	82 (11.9%)	56 (12.2%)	26 (11.3%)	
**SCWD classification^†^**				0.092
Non-sarcopenia	640 (92.9%)	421 (91.7%)	219 (95.2%)	
Sarcopenia	49 (7.1%)	38 (8.3%)	11 (4.8%)	
**FNIH classification^†^**				0.090
Non-sarcopenia	658 (95.5%)	434 (94.6%)	224 (97.4%)	
Sarcopenia	31 (4.5%)	25 (5.4%)	6 (2.6%)	

*HbA1c, glycated hemoglobin; FPG, fasting plasma glucose; 2hPG, 2-h postprandial glucose; HOMA-IR, homeostasis model assessment of insulin resistance; AST, aspartate aminotransferase; ALT, alanine aminotransferase; eGFR, estimated glomerular filtration rate; BMI, body mass index; WC, waist circumference; CC, calf circumference; HGS, handgrip strength; GS, gait speed; ASM, appendicular skeletal muscle mass; SMI, skeletal muscle mass index. The Student’s t-test and Mann–Whitney U-test were used for the continuous variables and the X^2^ test for the categorical variables.*

*Data are presented as the means (SDs).

*^†^Data are presented as numbers (percentages).*

*^‡^Data are presented as medians (interquartile ranges).*

*^a^The p-value represents the difference between the male and female groups.*

### Prevalence of Sarcopenia

Table 2 presents the incidence of sarcopenia according to the two screening instruments and the five diagnostic criteria. In the entire study population, the median scores for SARC-F and SARC-CalF were 0 and 3, respectively. Based on SARC-F and SARC-CalF, the prevalence of sarcopenia in our study population was 4.5 and 20.3%, respectively. According to the recommended cut-off values for various diagnostic criteria, the prevalence of sarcopenia was 19.2, 16.7, 11.9, 7.1, and 4.5%. The prevalence of sarcopenia varied between 5.4 and 21.1% in men and 2.6 and 15.2% in women. Irrespective of the reference benchmarks applied, sarcopenia was more common in men than in women; however, the differences were not significant.

### Comparison of SARC-F and SARC-CalF in the Whole Study Population

Table 3 presents the results of sensitivity/specificity analyses, as well as the AUC of SARC-F and SARC-CalF in the course of applying various diagnostic criteria as the reference benchmark. The sensitivity of the two tools varied in the following ranges: SARC-F, 61.4–67.4%; and SARC-ClaF, 82.6–91.8%. The values of the specificity ranges were as follows: SARC-F, 63.1–67.3%; and SARC-ClaF, 51.5–61.2%. The PPV outcomes ranged from 7.6% (for SARC-F against FNIH) to 33.5% (for SARC-CalF against EWGSOP1), while a deviation is recorded in NPV as it varies from 88.0% (for SARC-F against EWGSOP1) to 99.1% (for SARC-CalF against FNIH). Irrespective of the type of diagnostic criteria utilized as the reference standard, compared to the SARC-F, the SARC-CalF had more suitable sensitivity and lower specificity.

**TABLE 3:** Sensitivity, specificity, PPV, NPV, +LR, and −LR analyses and ROC curves for SARC-F and SARC-CalF validation against varying sarcopenia criteria.

	Sensitivity %	Specificity %	PPV %	NPV %	+LR	−LR	AUC	*p*-Value^a^
**EWGSOP1 classification**								
SARC-F	61.4 (52.5–69.7)	67.3 (63.3–71.2)	30.8 (27.1–34.8)	88.0 (85.5–90.2)	1.9 (1.6–2.2)	0.6 (0.5–0.7)	0.67 (0.63–0.71)	<0.001
SARC-CalF	82.6 (75.0–88.6)	61.2 (57.0–65.3)	33.5 (30.7–36.5)	93.7 (91.0–95.6)	2.1 (1.9–2.4)	0.3 (0.2–0.4)	0.79 (0.76–0.82)	
**AWGS2019 classification**								
SARC-F	62.6 (53.1–71.5)	66.7 (62.7–70.6)	27.4 (23.9–31.2)	89.9 (87.5–91.9)	1.9 (1.6–2.3)	0.6 (0.4–0.7)	0.67 (0.63–0.70)	<0.001
SARC-CalF	87.8 (80.4–93.2)	61.0 (56.8–65.0)	31.1 (28.5–33.8)	96.2 (93.8–97.6)	2.3 (2.0–2.5)	0.2 (0.1–0.3)	0.81 (0.78–0.84)	
**IWGS classification**								
SARC-F	63.4 (52.0–73.8)	65.2 (61.3–69.0)	19.8 (16.8–23.1)	93.0 (90.8–94.6)	1.8 (1.5–2.2)	0.6 (0.4–0.8)	0.65 (0.62–0.69)	<0.001
SARC-CalF	89.0 (80.2–94.9)	54.9 (50.8–58.9)	21.0 (19.2–23.0)	97.4 (95.2–98.6)	2.0 (1.8–2.2)	0.2 (0.1–0.4)	0.78 (0.74–0.81)	
**SCWD classification**								
SARC-F	67.4 (52.5–80.1)	64.1 (60.2–67.8)	12.5 (10.3–15.2)	96.2 (94.5–97.5)	1.9 (1.5–2.3)	0.5 (0.3–0.8)	0.66 (0.62–0.69)	<0.001
SARC-CalF	91.8 (80.4–97.7)	52.8 (48.9–56.7)	13.0 (11.7–14.3)	98.8 (97.1–99.5)	2.0 (1.7–2.2)	0.2 (0.1–0.4)	0.78 (0.75–0.81)	
**FNIH classification**								
SARC-F	64.5 (45.4–80.8)	63.1 (59.3–66.8)	7.6 (5.9–9.8)	97.4 (95.9–98.4)	1.8 (1.3–2.3)	0.6 (0.3–0.9)	0.65 (0.62–0.69)	0.052
SARC-CalF	90.3 (74.2–98.0)	51.5 (47.6–55.4)	8.1 (7.1–9.2)	99.1 (97.5–99.7)	1.9 (1.6–2.1)	0.2 (0.1–0.6)	0.74 (0.71–0.77)	

*PPV, positive predictive value; NPV, negative predictive value; +LR, positive likelihood ratio; −LR, negative likelihood ratio; AUC, area under the ROC curves. Values within parentheses represent the 95% confidential intervals.*

*^a^The p-value represents the difference between the SARC-F and SARC-CalF groups.*

**FIGURE 1:**
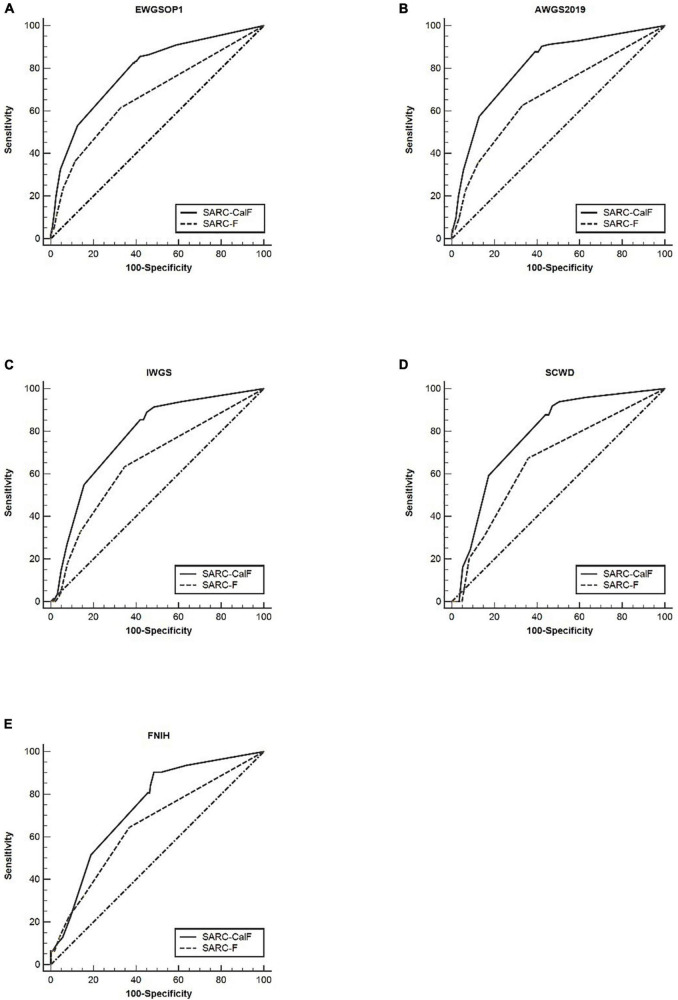
The ROC curves of SARC-F and SARC-CalF were contrasted with various reference standards in the entire population studied: **(A)** sarcopenia according to the EWGSOP1 criteria; **(B)** sarcopenia according to AWGS2019 criteria; **(C)** sarcopenia according to IWGS criteria; **(D)** sarcopenia according to SCWD criteria; and **(E)** sarcopenia according to FNIH criteria.

As shown in Figure 1, the ROC curves of the two screening tools against various standards in the entire study population are plotted. The ranges of AUCs of SARC-F and SARC-CalF are 0.65–0.67 and 0.74–0.81, respectively. Regarding the AUCs, unless the FNIH criteria were implemented, the variation between SARC-F and SARC-CalF was statistically significant (*p* < 0.001). When comparing the two screening tools, the SARC-CalF had the most significant AUC but only against the AWGS2019 criteria (0.81). These outcomes indicate that a nearly high level of diagnostic value was recorded. In contrast, the least AUC against the IWGS and FNIH criteria was recorded in SARC-F (0.65), and it likewise had a correspondingly small AUC for EWGSOP (0.67), AWGS2019 (0.67), and SCWD (0.66).

### Comparison of SARC-F and SARC-CalF in Each Sex

**FIGURE 2:**
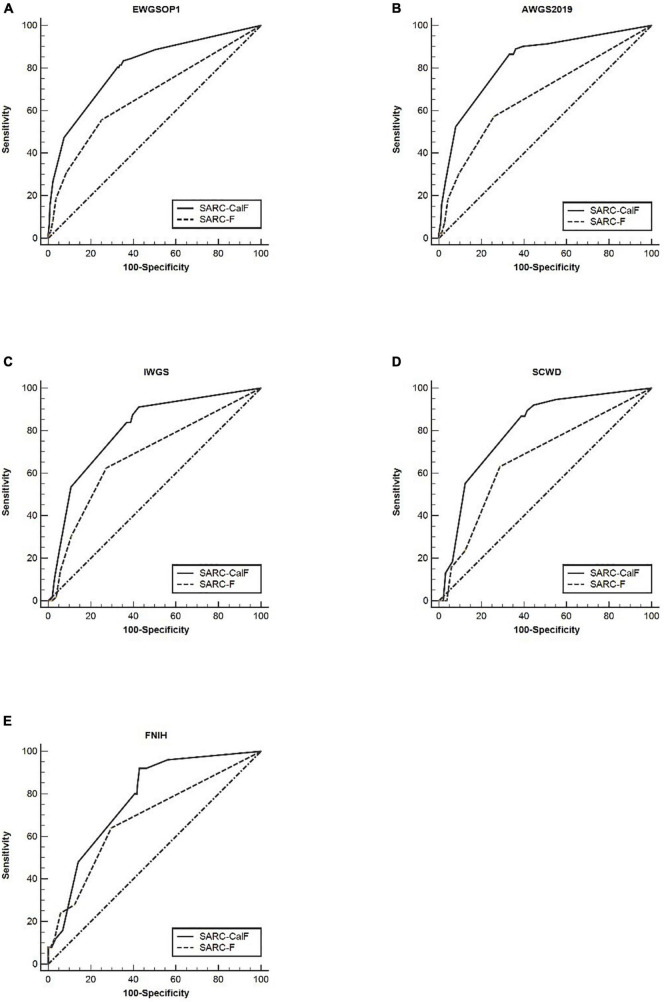
The ROC curves of SARC-F and SARC-CalF compared with various reference standards in men: **(A)** sarcopenia according to the EWGSOP1 criteria; **(B)** sarcopenia according to AWGS2019 criteria; **(C)** sarcopenia according to IWGS criteria; **(D)** sarcopenia according to SCWD criteria; and **(E)** sarcopenia according to FNIH criteria.

Table 4 presents the results of sensitivity/specificity analyses and AUCs of SARC-F and SARC-CalF in humans using various diagnostic criteria as reference standards. In men, SARC-CalF displayed more suitable sensitivity as a reference standard, regardless of diagnostic criteria; however, in contrast with SARC-F, it displays a lower specificity. For instance, using EWGSOP1 as a reference standard, the sensitivities of SARC-F and SARC-CalF were 55.7 and 83.5%, and the specificities were 74.9 and 64.6%, respectively. In Figure 2, the ROC curves for SARC-F and SARC-CalF against various reference standards in men are depicted. Through the application of the EWGSOP1 criteria, the respective AUC values for SARC-F and SARC-CalF were 0.67 and 0.80. Therefore, the difference was significant (*p* < 0.001). In this study, the corresponding outcomes were obtained using AWGS2019, IWGS, and SCWD. Assuming that the FNIH criteria were applied, there was no significant difference (*p* = 0.076).

**TABLE 4:** Sensitivity, specificity, PPV, NPV, +LR, and −LR analyses and ROC curves for SARC-F and SARC-CalF validation against different sarcopenia criteria in men.

	Sensitivity %	Specificity %	PPV %	NPV %	+LR	−LR	AUC	*p*-Value^a^
**EWGSOP1 classification**								
SARC-F	55.7 (45.2–65.8)	74.9 (70.1–79.2)	37.2 (31.6–43.3)	86.3 (83.3–88.8)	2.2 (1.7–2.8)	0.6 (0.5–0.7)	0.67 (0.62–0.71)	<0.001
SARC-CalF	83.5 (74.6–90.3)	64.6 (59.5–69.6)	38.8 (34.9–42.7)	93.6 (90.3–95.8)	2.4 (2.0–2.8)	0.3 (0.2–0.4)	0.80 (0.76–0.84)	
**AWGS2019 classification**								
SARC-F	57.3 (45.9–68.2)	74.0 (69.3–78.4)	32.4 (27.1–38.2)	88.9 (86.0–91.2)	2.2 (1.7–2.8)	0.6 (0.4–0.7)	0.67 (0.63–0.71)	<0.001
SARC-CalF	86.6 (77.3–93.1)	66.8 (61.8–71.6)	36.2 (32.5–40.2)	95.8 (92.9–97.6)	2.6 (2.2–3.1)	0.2 (0.1–0.3)	0.83 (0.79–0.86)	
**IWGS classification**								
SARC-F	62.5 (48.5–75.1)	72.7 (68.1–77.0)	24.1 (19.7–29.2)	93.3 (90.8–95.2)	2.3 (1.8–3.0)	0.5 (0.4–0.7)	0.68 (0.64–0.72)	<0.001
SARC-CalF	91.1 (80.4–97.0)	57.3 (52.3–62.2)	22.9 (20.5–25.4)	97.9 (95.2–99.1)	2.1 (1.9–2.5)	0.2 (0.1–0.4)	0.80 (0.77–0.84)	
**SCWD classification**								
SARC-F	63.2 (46.0–78.2)	71.3 (66.7–75.5)	16.6 (13.0–20.9)	95.5 (93.4–97.0)	2.2 (1.7–2.9)	0.5 (0.3–0.8)	0.67 (0.62–0.71)	<0.001
SARC-CalF	86.8 (71.9–95.6)	61.3 (56.4–66.0)	16.8 (14.6–19.4)	98.1 (95.8–99.2)	2.2 (1.9–2.7)	0.2 (0.1–0.5)	0.80 (0.76–0.84)	
**FNIH classification**								
SARC-F	64.0 (42.5–82.0)	70.3 (65.7–74.5)	11.0 (8.2–14.7)	97.1 (95.2–98.3)	2.2 (1.6–3.0)	0.5 (0.3–0.9)	0.68 (0.64–0.72)	0.076
SARC-CalF	92.0 (74.0–99.0)	57.1 (52.3–61.9)	11.0 (9.5–12.7)	99.2 (97.0–99.8)	2.2 (1.8–2.5)	0.2 (0.1–0.5)	0.77 (0.73–0.81)	

*PPV, positive predictive value; NPV, negative predictive value; +LR, positive likelihood ratio; −LR, negative likelihood ratio; AUC, area under the ROC curves. Values within parentheses represent the 95% confidential intervals.*

*^a^The p-value represents the difference between the SARC-F and SARC-CalF groups.*

**FIGURE 3:**
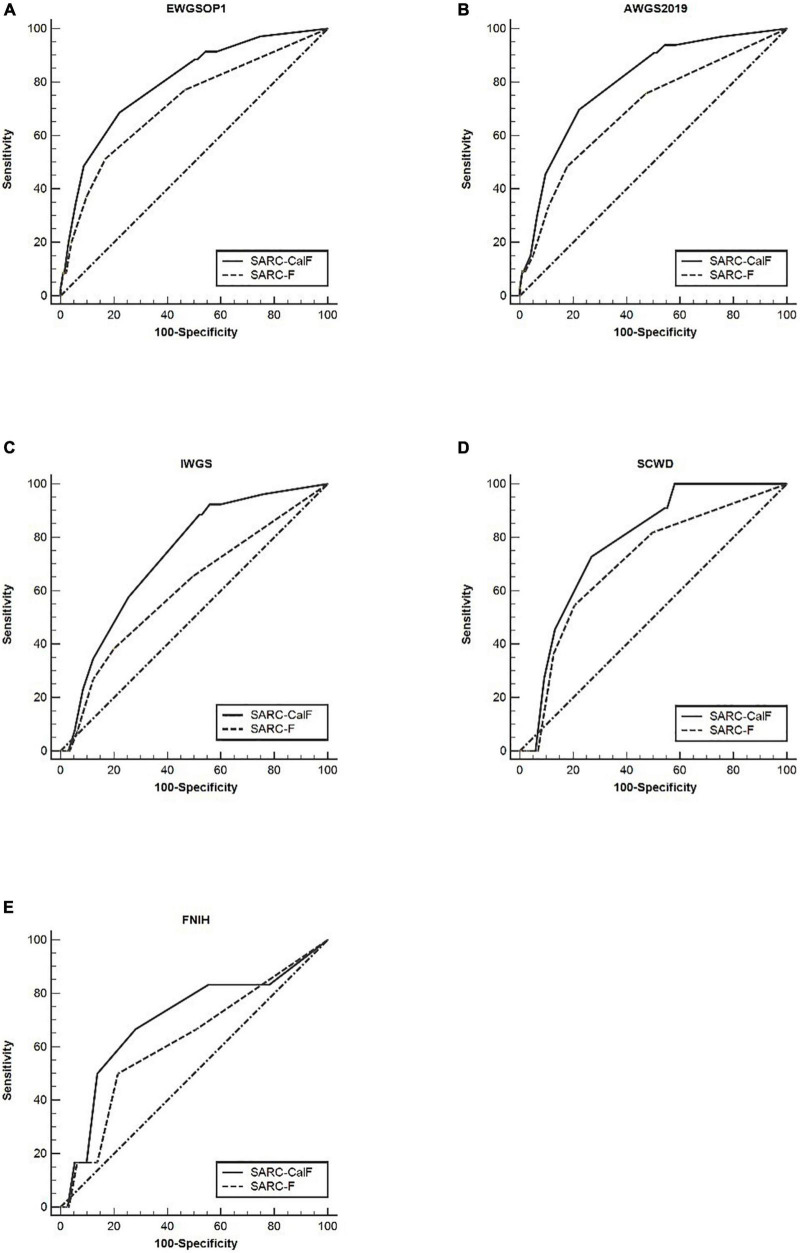
The ROC curves of SARC-F and SARC-CalF compared with various reference standards in women: **(A)** sarcopenia according to the EWGSOP1 criteria; **(B)** sarcopenia according to AWGS2019 criteria; **(C)** sarcopenia according to IWGS criteria; **(D)** sarcopenia according to SCWD criteria; and **(E)** sarcopenia according to FNIH criteria.

Table 5 presents the results of sensitivity/specificity analyses, as well as the AUCs of SARC-F and SARC-CalF in women with diverse diagnostic criteria serving as a reference benchmark. Regardless of the adopted reference standard utilized for women, SARC-CalF equally demonstrated more suitable sensitivity and identical specificity in contrast to SARC-F. For instance, when using EWGSOP1 as the reference standard, the sensitivities of SARC-F and SARC-CalF were 51.4 and 68.6%, and the specificities were 83.1 and 78.0%, respectively. The ROC curves for SARC-F and SARC-CalF against various reference standards in women are shown in Figure 3. Using the EWGSOP1 criteria, the AUCs for SARC-F and SARC-CalF were 0.72 and 0.80, respectively. There was a significant difference between the groups (*p* = 0.025). With the assistance of AWGS2019 and IWGS, similar results were obtained. The AUC of SARC-CalF is moderate when applied to different sensitivities to various reference benchmarks when applied for different sexes.

**TABLE 5:** Analysis of the curves of sensitivity, specificity, PPV, VPN, +LR, and −LR and ROC for the validation of SARC-F and SARC-CalF against various criteria of sarcopenia in women.

	Sensitivity %	Specificity %	PPV %	NPV %	+LR	−LR	AUC	*p*-Value^a^
**EWGSOP1 classification**								
SARC-F	51.4 (34.0–68.6)	83.1 (77.1–88.1)	35.3 (25.9–46.0)	90.5 (87.1–93.1)	3.0 (1.9–4.8)	0.6 (0.4–0.8)	0.72 (0.65–0.77)	0.025
SARC-CalF	68.6 (50.7–83.1)	78.0 (71.5–83.6)	35.8 (28.3–44.1)	93.3 (89.4–95.8)	3.1 (2.2–4.4)	0.4 (0.2–0.7)	0.80 (0.74–0.85)	
**AWGS2019 classification**								
SARC-F	48.5 (30.8–66.5)	82.2 (76.2–87.3)	31.4 (22.4–42.1)	90.5 (87.2–93.0)	2.7 (1.7–4.3)	0.6 (0.4–0.9)	0.70 (0.63–0.75)	0.005
SARC-CalF	69.7 (51.3–84.4)	77.7 (71.2–83.3)	34.3 (27.0–42.4)	93.9 (90.1–96.3)	3.1 (2.2–4.4)	0.4 (0.2–0.7)	0.80 (0.74–0.85)	
**IWGS classification**								
SARC-F	38.5 (20.2–59.4)	79.9 (73.7–85.2)	19.6 (12.3–29.9)	91.1 (88.2–93.3)	1.9 (1.1–3.3)	0.8 (0.6–1.1)	0.61 (0.54–0.67)	0.006
SARC-CalF	88.5 (69.8–97.6)	48.0 (41.0–55.1)	17.8 (15.2–20.8)	97.0 (91.8–99.0)	1.7 (1.4–2.1)	0.2 (0.1–0.7)	0.73 (0.67–0.79)	
**SCWD classification**								
SARC-F	54.6 (23.4–83.3)	79.5 (73.5–84.6)	11.8 (6.8–19.5)	97.2 (94.8–98.5)	2.7 (1.5–4.8)	0.6 (0.3–1.1)	0.70 (0.64–0.76)	0.248
SARC-CalF	72.7 (39.0–94.0)	73.1 (66.7–78.8)	11.9 (8.2–17.1)	98.2 (95.3–99.3)	2.7 (1.8–4.1)	0.4 (0.1–1.0)	0.78 (0.72–0.83)	
**FNIH classification**								
SARC-F	50.0 (11.8–88.2)	78.6 (72.6–83.8)	5.9 (2.6–12.6)	98.3 (96.3–99.2)	2.3 (1.0–5.4)	0.6 (0.3–1.4)	0.62 (0.56–0.69)	0.227
SARC-CalF	66.7 (22.3–95.7)	71.9 (65.5–77.7)	6.0 (3.4–10.4)	98.8 (96.3–99.6)	2.4 (1.3–4.3)	0.5 (0.1–1.4)	0.70 (0.64–0.76)	

*PPV, positive predictive value; NPV, negative predictive value; +LR, positive likelihood ratio; −LR, negative likelihood ratio; AUC, area under the ROC curves. Values within parentheses represent the 95% confidential intervals.*

*^a^The p-value represents the difference between the SARC-F and SARC-CalF groups.*

### Comparison of SARC-F and SARC-CalF in Each Age Group

**FIGURE 4:**
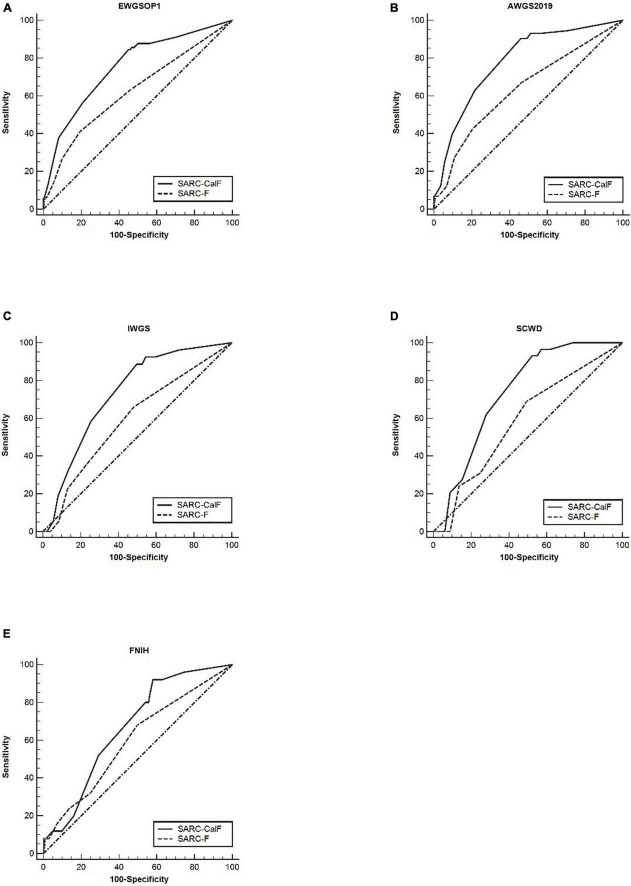
The ROC curves of SARC-F and SARC-CalF compared with various reference norms in the older group: **(A)** sarcopenia according to the EWGSOP1 criteria; **(B)** sarcopenia according to AWGS2019 criteria; **(C)** sarcopenia according to IWGS criteria; **(D)** sarcopenia according to SCWD criteria; and **(E)** sarcopenia according to FNIH criteria.

**FIGURE 5:**
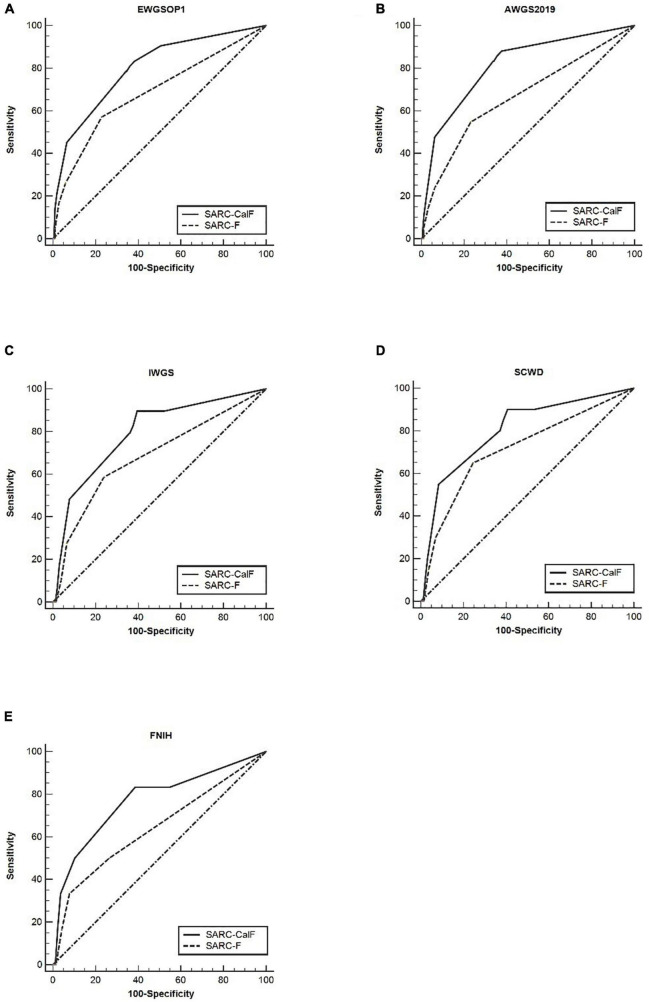
The ROC curves of SARC-F and SARC-CalF compared with various reference standards in the category of younger subjects: **(A)** sarcopenia according to the EWGSOP1 criteria; **(B)** sarcopenia according to AWGS2019 criteria; **(C)** sarcopenia according to IWGS criteria; **(D)** sarcopenia according to SCWD criteria; and **(E)** sarcopenia according to FNIH criteria.

Table 6 presents the screening potential of SARC-F alongside SARC-CalF in subjects aged ≥60 years during the application of various diagnostic criteria as reference standards. Sensitivity/specificity analyses showed related results in the older group, in contrast to the entire study population. The sensitivity of the two instruments varied in the following areas: SARC-F, 41.1–69.0%; and SARC-CalF, 84.4–93.1%. The ranges of specificity were as follows: SARC-F, 50.3–80.7%; and SARC-CalF, 42.0–54.9%. As shown in Figure 4, during the process of applying the EWGSOP1, AWGS2019, IWGS, and SCWD, the variation in AUC between SARC-F and SARC-CalF was generally significant. Table 7 presents the screening potentials of SARC-F and SARC-CalF in subjects aged <60 years with varying diagnostic criteria as a reference standard. In the younger group, the sensitivity of the two instruments varied in the following areas: SARC-F, 33.3–65.0%; and SARC-Calf, 83.3–90.0%. The ranges of specificity were as follows: SARC-F, 75.4–92.2%; and SARC-CalF, 59.3–62.4%. As shown in Figure 5, there is a significant variation in AUC between SARC-F and SARC-CalF when the EWGSOP1, AWGS2019, and IWGS criteria were applied.

**TABLE 6:** Sensitivity, specificity, PPV, VPN, +LR, and −LR tests and ROC curves for SARC-F and SARC-CalF validation against various sarcopenia criteria in the older group.

	Sensitivity %	Specificity %	PPV %	NPV %	+LR	−LR	AUC	*p*-Value^a^
**EWGSOP1 classification**								
SARC-F	41.1 (30.8–52.0)	80.7 (75.0–85.6)	45.1 (36.4–54.1)	78.0 (74.7–81.0)	2.1 (1.5–3.1)	0.7 (0.6–0.9)	0.63 (0.57–0.68)	<0.001
SARC-CalF	84.4 (75.3–91.2)	54.9 (48.3–61.4)	42.0 (38.0–46.1)	90.1 (84.8–93.8)	1.9 (1.6–2.2)	0.3 (0.2–0.5)	0.75 (0.70–0.80)	
**AWGS2019 classification**								
SARC-F	42.5 (31.0–54.6)	79.6 (74.1–84.4)	37.8 (29.7–46.6)	82.6 (79.4–85.4)	2.1 (1.4–3.0)	0.7 (0.6–0.9)	0.64 (0.58–0.69)	<0.001
SARC-CalF	90.4 (81.2–96.1)	54.0 (47.6–60.3)	36.5 (33.0–40.1)	95.1 (90.4–97.5)	2.0 (1.7–2.3)	0.2 (0.1–0.4)	0.78 (0.74–0.83)	
**IWGS classification**								
SARC-F	66.0 (51.7–78.5)	51.9 (45.7–57.9)	21.2 (17.6–25.3)	88.6 (84.0–92.0)	1.4 (1.1–1.7)	0.7 (0.4–1.0)	0.60 (0.54–0.65)	<0.001
SARC-CalF	88.7 (77.0–95.7)	50.4 (44.2–56.5)	26.0 (23.1–29.0)	95.8 (91.4–98.0)	1.8 (1.5–2.1)	0.2 (0.1–0.5)	0.73 (0.68–0.78)	
**SCWD classification**								
SARC-F	69.0 (49.2–84.7)	50.7 (44.8–56.5)	12.1 (9.5–15.3)	94.3 (90.5–96.6)	1.4 (1.1–1.8)	0.6 (0.4–1.1)	0.59 (0.53–0.64)	<0.001
SARC-CalF	93.1 (77.2–99.2)	47.6 (41.8–53.5)	14.9 (13.1–16.9)	98.6 (94.8–99.6)	1.8 (1.5–2.1)	0.2 (0.1–0.6)	0.73 (0.68–0.78)	
**FNIH classification**								
SARC-F	68.0 (46.5–85.1)	50.3 (44.5–56.2)	10.3 (7.9–13.3)	94.9 (91.3–97.1)	1.4 (1.0–1.8)	0.6 (0.4–1.1)	0.60 (0.54–0.65)	0.195
SARC-CalF	92.0 (74.0–99.0)	42.0 (36.3–47.8)	11.7 (10.3–13.4)	98.4 (94.3–99.6)	1.6 (1.4–1.8)	0.2 (0.1–0.7)	0.67 (0.61–0.72)	

*PPV, positive predictive value; NPV, negative predictive value; +LR, positive likelihood ratio; −LR, negative likelihood ratio; AUC, area under the ROC curves. Values within parentheses represent the 95% confidential intervals.*

*^a^The p-value represents the difference between the SARC-F and SARC-CalF groups.*

**TABLE 7:** Sensitivity, specificity, PPV, NPV, +LR, and −LR assessments and ROC curves for SARC-F and SARC-CalF validation against various criteria of sarcopenia in the group comprising of the younger group.

	Sensitivity %	Specificity %	PPV %	NPV %	+LR	−LR	AUC	*p*-Value^a^
**EWGSOP1 classification**								
SARC-F	57.1 (41.0–72.3)	77.2 (72.2–81.6)	24.5 (18.9–31.1)	93.3 (90.7–95.2)	2.5 (1.8–3.5)	0.6 (0.4–0.8)	0.69 (0.64–0.73)	0.005
SARC-CalF	83.3 (68.6–93.0)	61.7 (56.2–67.0)	22.0 (18.9–25.5)	96.6 (93.5–98.3)	2.2 (1.8–2.6)	0.3 (0.1–0.5)	0.80 (0.76–0.84)	
**AWGS2019 classification**								
SARC-F	54.8 (38.7–70.2)	76.9 (71.9–81.3)	23.5 (17.9–30.1)	92.9 (90.3–94.8)	2.4 (1.7–3.3)	0.6 (0.4–0.8)	0.67 (0.62–0.72)	<0.001
SARC-CalF	88.1 (74.4–96.0)	62.4 (56.8–67.6)	23.3 (20.2–26.6)	97.6 (94.6–98.9)	2.3 (2.0–2.8)	0.2 (0.1–0.4)	0.82 (0.77–0.85)	
**IWGS classification**								
SARC-F	58.6 (38.9–76.5)	76.0 (71.0–80.4)	17.3 (12.8–23.1)	95.5 (93.2–97.1)	2.4 (1.7–3.5)	0.5 (0.4–0.8)	0.68 (0.63–0.73)	0.017
SARC-CalF	89.7 (72.6–97.8)	60.5 (55.1–65.8)	16.4 (14.0–19.0)	98.6 (95.9–99.5)	2.3 (1.9–2.7)	0.2 (0.1–0.5)	0.79 (0.75–0.83)	
**SCWD classification**								
SARC-F	65.0 (40.8–84.6)	75.4 (70.5–79.9)	13.3 (9.5–18.1)	97.4 (95.3–98.5)	2.7 (1.8–3.8)	0.5 (0.3–0.8)	0.72 (0.67–0.76)	0.090
SARC-CalF	90.0 (68.3–98.8)	59.3 (53.9–64.5)	11.3 (9.5–13.4)	99.0 (96.5–99.7)	2.2 (1.8–2.7)	0.2 (0.1–0.6)	0.80 (0.76–0.84)	
**FNIH classification**								
SARC-F	33.3 (4.3–77.7)	92.2 (89.0–94.8)	6.7 (2.1–19.0)	98.8 (97.9–99.3)	4.3 (1.3–14.0)	0.7 (0.4–1.3)	0.64 (0.59–0.69)	0.143
SARC-CalF	83.3 (35.9–99.6)	61.4 (56.1–66.4)	3.5 (2.4–5.0)	99.5 (97.4–99.9)	2.2 (1.5–3.2)	0.3 (0.1–1.6)	0.77 (0.72–0.81)	

*PPV, positive predictive value; NPV, negative predictive value; +LR, positive likelihood ratio; −LR, negative likelihood ratio; AUC, area under the ROC curves. Values within parentheses represent the 95% confidential intervals.*

*^a^The p-value represents the difference between the SARC-F and SARC-CalF groups.*

In the category of older subjects, the AUC of the two instruments was from 0.59 to 0.78, while it was from 0.64 to 0.82 in younger subjects. Therefore, a comparison of both groups demonstrated that the AUC of the two screening tools was higher in the younger subjects than in the older subjects. Furthermore, it was observed that the increase in age is directly proportional to the increase in sensitivity to SARC-CalF. In contrast, a contradictory trend was demonstrated in the specificity of SARC-CalF.

## Discussion

This study showed that SARC-CalF was significantly more suitable than SARC-F in sarcopenia screening for patients with T2DM in terms of sensitivity and general diagnostic accuracy using varying criteria as a gold benchmark. Since sarcopenia is associated with serious consequences on the health of older subjects, early recommendation of preventive strategies is highly necessary. Thus, from a clinical perspective, it is essential to provide an early diagnosis using simple and effective sarcopenia screening tools, preferably, a screening tool exhibiting high sensitivity while simultaneously maintaining high specificity (23).

Based on the general consensus about sarcopenia, for diagnosis confirmation, it has been demonstrated that low muscle mass and function are essential. SARC-F is the first screening tool for sarcopenia and has been widely applied in the field of sarcopenia. Thus far, the validation of the research has been implemented in China (8, 12, 24), America (25), Japan (26), Turkey (10), and Brazil (27, 28), to mention a few. Nevertheless, previous studies have revealed that it has high specificity and low sensitivity. A novel study conducted by Parra-Rodríguez et al. revealed that in 487 men and women, the sensitivity to SARC-F was 35.6% with a specificity of 82.2% (9). The outcome of this study corresponds to the results of Woo et al., which revealed that the sensitivity and specificity of SARC-F were 9.9 and 94.4%, respectively (8). Thus, due to the low sensitivity of SARC-F, it has subsequently brought about limitations to its clinical application.

From the findings of Kawakami et al., a positive correlation was found between CC and muscle mass (29). Furthermore, the SARC-CalF, integrating CC and SARC-F, was developed by Barbosa-Silva. This research, which involved 179 older people in communities in Brazil, found that the sensitivity and specificity of SARC-CalF were 66.7 and 82.9%, respectively (11). Following the application of the same standard, it was reported by Yang et al. that SARC-CalF with sensitivity and specificity in Chinese nursing home occupants was 58.9 and 84.5%, respectively (13). Therefore, it was proposed by the above researchers that SARC-CalF should be utilized as a modified version for enhancing the sensitivity of SARC-F (11). In this study, the sensitivity and specificity displayed by SARC-CalF were 82.6 and 61.2%, respectively, by applying the EWGSOP1 criteria. In contrast to past research, this study showed relatively lower specificity, albeit a higher sensitivity, possibly as a result of the varying clinical features (race, sex, and age) of the subjects. In addition, all subjects in the study have diabetes. Applying various diagnostic criteria, the AUC of SARC-F ranged from 0.65 to 0.67, and the AUC of SARC-CalF ranged from 0.74 to 0.81. Thus, SARC-F displayed low diagnostic precision, and SARC-CalF demonstrated moderate diagnostic accuracy. In general, with regard to the screening for sarcopenia in adults with T2DM, SARC-CalF is more effective than SARC-F.

This study demonstrated that sex and age could affect the screening potential of the two screening tools. Overall, men tend to overestimate their physical abilities (30), while women could underestimate their physical abilities because of their diverse perceptions. The results of this study showed that SARC-F and SARC-CalF have significantly higher values in women than in men. Regarding sex, SARC-CalF in men showed higher sensitivity and AUC compared with women. This result in contrast to the conclusion of Mo et al. (31). Moreover, age was one of the criteria. In general, the specificity and AUC of SARC-CalF decreased with age. In contrast, the trend displayed by the sensitivity of SARC-CalF showed the opposite trend. This result corroborate the study by Mo et al. (31).

Calf circumference tends to be profoundly impacted by obesity and edema, which may mask sarcopenia (13, 32). Nevertheless, various optimal cut-off points exist for CC in various ethnic groups. Kawakami et al. utilized 34 and 33 cm as cut-off points for CC in Japanese men and women, respectively, in estimating low muscle mass (29). In a Turkish study, 33 cm was proposed as the threshold point for CC in men and women (33). In a recent study conducted by Hwang et al. in Taiwan, sections were recorded at 33 and 32 cm to predict sarcopenia in men and women, respectively. In our study, the median CC in men and women was 34 and 33 cm, respectively. Thus, the cut-off values of the screening recommended by the AWGS2019 consensus are appropriate for subjects utilized in this study.

Recent studies have reported that the CC *per se* was better than the SARC-F and SARC-CalF (31, 34, 35). Our data demonstrated that although both the SARC-CalF and CC outperformed the SARC-F, demonstrating moderate diagnostic accuracy; the CC *per se* was not significantly better than the SARC-CalF. In contrast, the SARC-CalF was slightly more sensitive than the CC. This finding should be addressed in future studies with larger sample sizes. Overall, the conclusions remain unchanged. These data are shown in Supplementary Table 2. Moreover, a novel tool, known as the mini sarcopenia risk assessment (MSRA) questionnaire, was developed by Rossi et al., with sensitivity and specificity of 80.4 and 60.4%, respectively. The MSRA includes a comprehensive evaluation and nutritional assessment (36). This scale was validated in the study by Ming-Yang et al. in various populations and subsequently surmised that MSRA-5 could act as a dependable and valid tool for screening sarcopenia (13, 37). Through the application of age, as well as BMI, Kurita et al. enhanced SARC-F and named this modified version SARC-F + EBM (38). In 2019, research conducted on 959 hospitalized Japanese patients showed that SARC-F + EBM had higher sensitivity than SARC-F, with a sensitivity of 77.8% and a specificity of 69.6% (38). Thus, the validation and comparison of these new screening tools in adults with T2DM should be evaluated in subsequent studies.

Certain drawbacks of this study need to be addressed. First, this study focused initially only on hospitalized patients with T2DM. Thus, this study outcome may not be appropriate for older people residing in the community. Second, cognitive functions were not assessed because our subjects were relatively young. Notwithstanding, SARC-F was developed based on the elderly population. Third, the prognostic value of SARC-F and SARC-CalF for adverse outcomes should be considered in future prospective studies as a cross-sectional study.

## Conclusion

In summary, early detection and intervention of sarcopenia are crucial. Regardless of the reference standard, sex, and age, SARC-CalF displayed more suitable sensitivity and diagnostic performance than SARC-F. Thus, SARC-CalF appears to be a more appropriate screening tool for sarcopenia in adults with T2DM. Subsequent research is needed to validate the utility of SARC-CalF in various populations and frameworks.

## Standard Biosecurity and Institutional Safety Procedures

All institutional security procedures were followed and biosecurity metrics were implemented. The hospital laboratory utilized for this research has a biosafety level 2 (BSL-2) standard, implying that all standards and protocols have been implemented in accordance with the guidelines of the Clinical and Laboratory Standards Institute (CLSI).

## Data Availability Statement

The raw data supporting the conclusions of this article will be made available by the authors, without undue reservation.

## Ethics Statement

The studies involving human participants were reviewed and approved by the Ethics Committee of The First Affiliated Hospital of Wenzhou Medical University. The patients/participants provided their written informed consent to participate in this study.

## Author Contributions

HZ conceived and designed this manuscript. JJ, ZZ, and YC conducted the data collection and participants’ recruiting exercises. HZ and ZX conducted the data analysis and interpretation. ZX and PZ prepared the manuscript. All authors in this study contributed to the article and subsequently approved the submitted version.

## Conflict of Interest

The authors declare that the research was conducted in the absence of any commercial or financial relationships that could be construed as a potential conflict of interest.

## Publisher’s Note

All claims expressed in this article are solely those of the authors and do not necessarily represent those of their affiliated organizations, or those of the publisher, the editors and the reviewers. Any product that may be evaluated in this article, or claim that may be made by its manufacturer, is not guaranteed or endorsed by the publisher.
