# Supplementary material for: Comparing SARC-CalF With SARC-F for Screening Sarcopenia in Adults With Type 2 Diabetes Mellitus
Source: Front Nutr. 2022 Mar 31;9:803924. doi: 10.3389/fnut.2022.803924 (PMC9009513; doi:10.3389/fnut.2022.803924)
Supplement: Supplementary file 1 [file Table_1.DOCX]

**Supplementary Table 1** The SARC-F and SARC-CalF Scales

| Components | Questions | SARC-F Score | SARC-CalF Score |
| --- | --- | --- | --- |
| Strength | Did you experience any difficulty in lifting or carrying 10 pounds? | None = 0 Some = 1 Great difficulty or unable to lift = 2 | None = 0 Some = 1 Great difficulty or unable to lift = 2 |
| Assistance in walking | Did you experience any difficulty in walking across a room? | None = 0 Some = 1 Great difficulty, use aids, or unable to walk = 2 | None = 0 Some = 1 Great difficulty, use aids, or unable to walk = 2 |
| Rising from a chair | Did you experience any difficulty in transferring from a chair or bed? | None = 0 Some = 1 Great difficulty or unable to transfer without help = 2 | None = 0 Some = 1 Great difficulty or unable to transfer without help = 2 |
| Climbing stairs | Did you experience any difficulty in climbing a flight of 10 steps? | None = 0 Some = 1 Great difficulty or unable to climb = 2 | None = 0 Some = 1 Great difficulty or unable to climb = 2 |
| Falls | Did you experience any falls in the past year? | None = 0 1–3 falls = 1 4 or more falls = 2 | None = 0 1–3 falls = 1 4 or more falls = 2 |
| Calf circumference |  | - | Females: > 33cm = 0 ≤ 33cm = 10 Males: >34cm = 0 ≤34cm =10 |

**Supplementary Table 2** Sensitivity, specificity, PPV, NPV, +LR, and -LR analyses and ROC curves for SARC-F, SARC-CalF, and CC validation against varying sarcopenia criteria.

|  | **Sensitivity%** | **Specificity%** | **PPV%** | **NPV%** | **+LR** | **-LR** | **AUC** |
| --- | --- | --- | --- | --- | --- | --- | --- |
| EWGSOP classification |  |  |  |  |  |  |  |
| CC | 84.2(75.6-90.7) | 68.0(63.0-72.7) | 41.5(37.4-45.6) | 94.1(91.0-96.2) | 2.6(2.2-3.1) | 0.2(0.1-0.4) | 0.83(0.79-0.86)^b^ |
| SARC-F | 61.4(52.5-69.7) | 67.3(63.3-71.2) | 30.8(27.1-34.8) | 88.0(85.5-90.2) | 1.9(1.6-2.2) | 0.6(0.5-0.7) | 0.67(0.63-0.71)^a,c^ |
| SARC-CalF | 82.6(75.0-88.6) | 61.2(57.0-65.3) | 33.5(30.7-36.5) | 93.7(91.0-95.6) | 2.1(1.9-2.4) | 0.3(0.2-0.4) | 0.79(0.76-0.82)^b^ |
| AWGS classification |  |  |  |  |  |  |  |
| CC | 71.3(60.6-80.5) | 85.4(81.4-88.7) | 52.1(45.3-58.9) | 93.0(90.5-94.9) | 4.9(3.7-6.4) | 0.3(0.2-0.5) | 0.85(0.82-0.88)^b^ |
| SARC-F | 62.6(53.1-71.5) | 66.7(62.7-70.6) | 27.4(23.9-31.2) | 89.9(87.5-91.9) | 1.9(1.6-2.3) | 0.6(0.4-0.7) | 0.67(0.63-0.70)^a,c^ |
| SARC-CalF | 87.8(80.4-93.2) | 61.0(56.8-65.0) | 31.1(28.5-33.8) | 96.2(93.8-97.6) | 2.3(2.0-2.5) | 0.2(0.1-0.3) | 0.81(0.78-0.84)^b^ |
| IWGS classification |  |  |  |  |  |  |  |
| CC | 90.3(80.1-96.4) | 64.0(59.2-68.6) | 27.3(24.4-30.4) | 97.8(95.4-99.0) | 2.5(2.2-2.9) | 0.2(0.1-0.3) | 0.82(0.78-0.85)^b^ |
| SARC-F | 63.4(52.0-73.8) | 65.2(61.3-69.0) | 19.8(16.8-23.1) | 93.0(90.8-94.6) | 1.8(1.5-2.2) | 0.6(0.4-0.8) | 0.65(0.62-0.69)^a,c^ |
| SARC-CalF | 89.0(80.2-94.9) | 54.9(50.8-58.9) | 21.0(19.2-23.0) | 97.4(95.2-98.6) | 2.0(1.8-2.2) | 0.2(0.1-0.4) | 0.78(0.74-0.81)^b^ |
| SCWD classification |  |  |  |  |  |  |  |
| CC | 83.8(68.0-93.8) | 80.0(75.9-83.6) | 26.1(21.8-30.8) | 98.3(96.6-99.2) | 4.2(3.3-5.3) | 0.2(0.1-0.4) | 0.86(0.82-0.89)^b,a^ |
| SARC-F | 67.4(52.5-80.1) | 64.1(60.2-67.8) | 12.5(10.3-15.2) | 96.2(94.5-97.5) | 1.9(1.5-2.3) | 0.5(0.3-0.8) | 0.66(0.62-0.69)^a,c^ |
| SARC-CalF | 91.8(80.4-97.7) | 52.8(48.9-56.7) | 13.0(11.7-14.3) | 98.8(97.1-99.5) | 2.0(1.7-2.2) | 0.2(0.1-0.4) | 0.78(0.75-0.81)^b,c^ |
| FNIH classification |  |  |  |  |  |  |  |
| CC | 77.3(54.6-92.2) | 67.0(62.4-71.3) | 10.2(8.0-12.8) | 98.4(96.6-99.2) | 2.3(1.8-3.0) | 0.3(0.2-0.7) | 0.74(0.70-0.78) |
| SARC-F | 64.5(45.4-80.8) | 63.1(59.3-66.8) | 7.6(5.9-9.8) | 97.4(95.9-98.4) | 1.8(1.3-2.3) | 0.6(0.3-0.9) | 0.65(0.62-0.69) |
| SARC-CalF | 90.3(74.2-98.0) | 51.5(47.6-55.4) | 8.1(7.1-9.2) | 99.1(97.5-99.7) | 1.9(1.6-2.1) | 0.2(0.1-0.6) | 0.74(0.71-0.77) |

PPV, positive predictive value; NPV, negative predictive value; +LR, positive likelihood ratio; -LR, negative likelihood ratio; AUC, the area under ROC curves.

Values within parentheses are the 95% confidential intervals.

^a^ Significantly different with SARC-CalF (*P* < 0.05)

^b^ Significantly different with SARC-F (*P* < 0.05)

^c^ Significantly different with CC (*P* < 0.05)
